# Supplementary material for: Cryo-EM structure of Chlamydomonas reinhardtii Photosystem I complexed with cytochrome c6
Source: Nat Commun. 2026 Mar 27;17:3031. doi: 10.1038/s41467-026-70944-9 (PMC13036084; doi:10.1038/s41467-026-70944-9)
Supplement: Supplementary file 1 — Supplementary Information [file 41467_2026_70944_MOESM1_ESM.pdf]

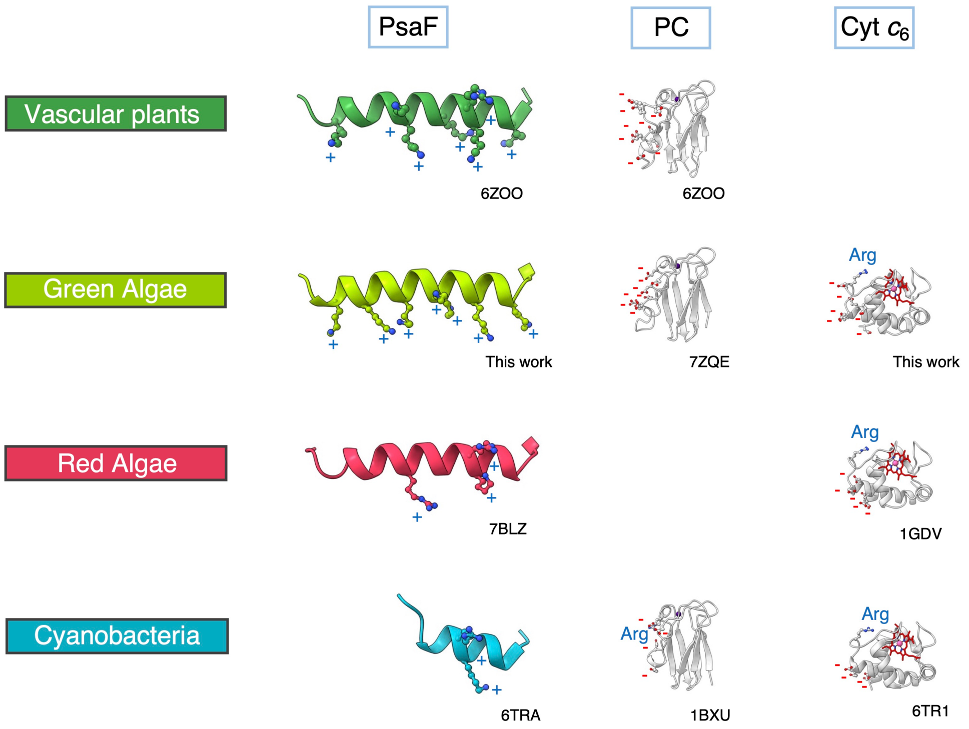

Supplementary Fig. 1. Comparison of the charged residues on binding interfaces of PsaF, Pc and Cyt c_6 from vascular plants, green algae, red algae and cyanobacteria. Positively and negatively charged residues are indicated by "+" and "-", respectively. The "Arg" represents the key residue in ancestral donors, which corresponds to *Chlamydomonas* Cyt c_6-R66. Each PDB ID is provided.

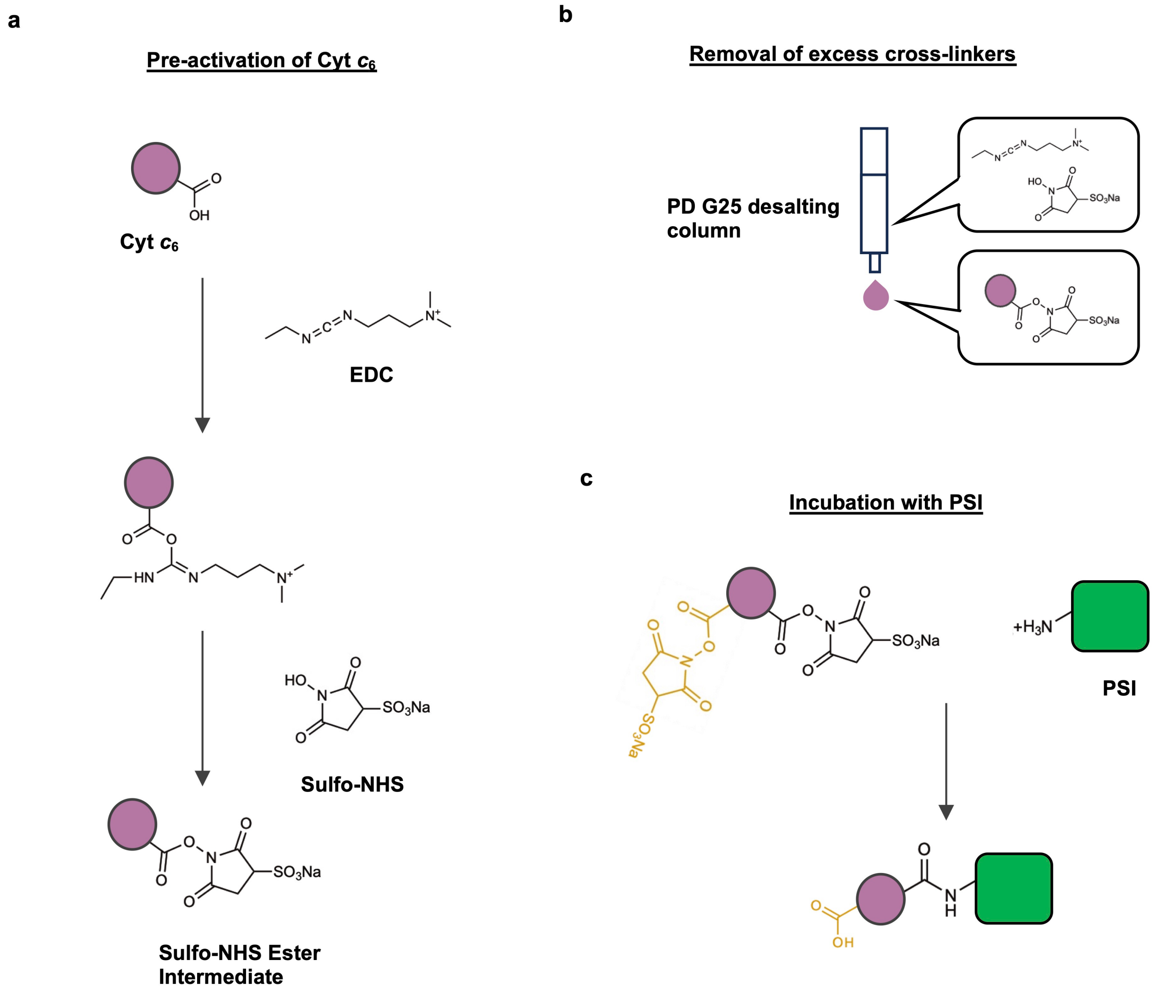

Supplementary Fig. 2. Schematic flow of Cyt c_6: PSI cross-linking reactions. **a**, Pre-activation of Cyt c_6. Incubation of Cyt c_6 with EDC and sulfo-NHS converts the carboxyl groups of Cyt c_6 into sulfo-NHS ester intermediates. **b**, Removal of excess cross-linkers. The activated Cyt c_6 is isolated from excess cross-linkers using PD G25 desalting columns. **c**, Incubation with PSI. The activated Cyt c_6 is thought to carry several sulfo-NHS esters. Those located in close proximity to amino groups of PSI (black) within Cyt c_6:PSI complexes form amido bonds, whereas the remaining esters (light brown) rapidly hydrolyze without generating cross-links.

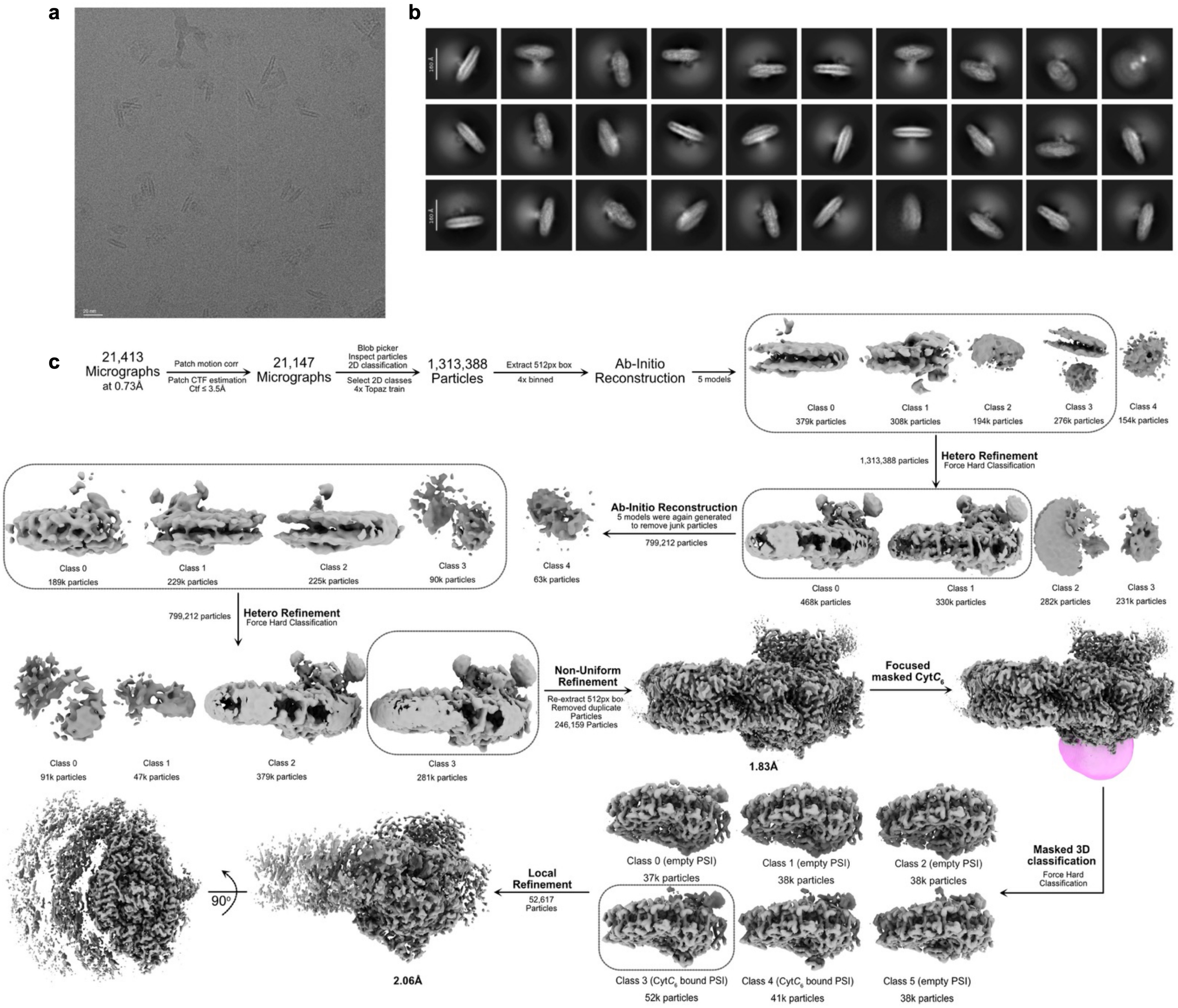

Supplementary Fig. 3. Cryo-EM data collection and processing workflow. **a**, A representative micrograph showing Cyt c_6:PSI particles. **b**, Reference-free 2D class averages revealing different views of the complex. **c**, Overview of the cryo-EM data processing pipeline performed in cryoSPARC.

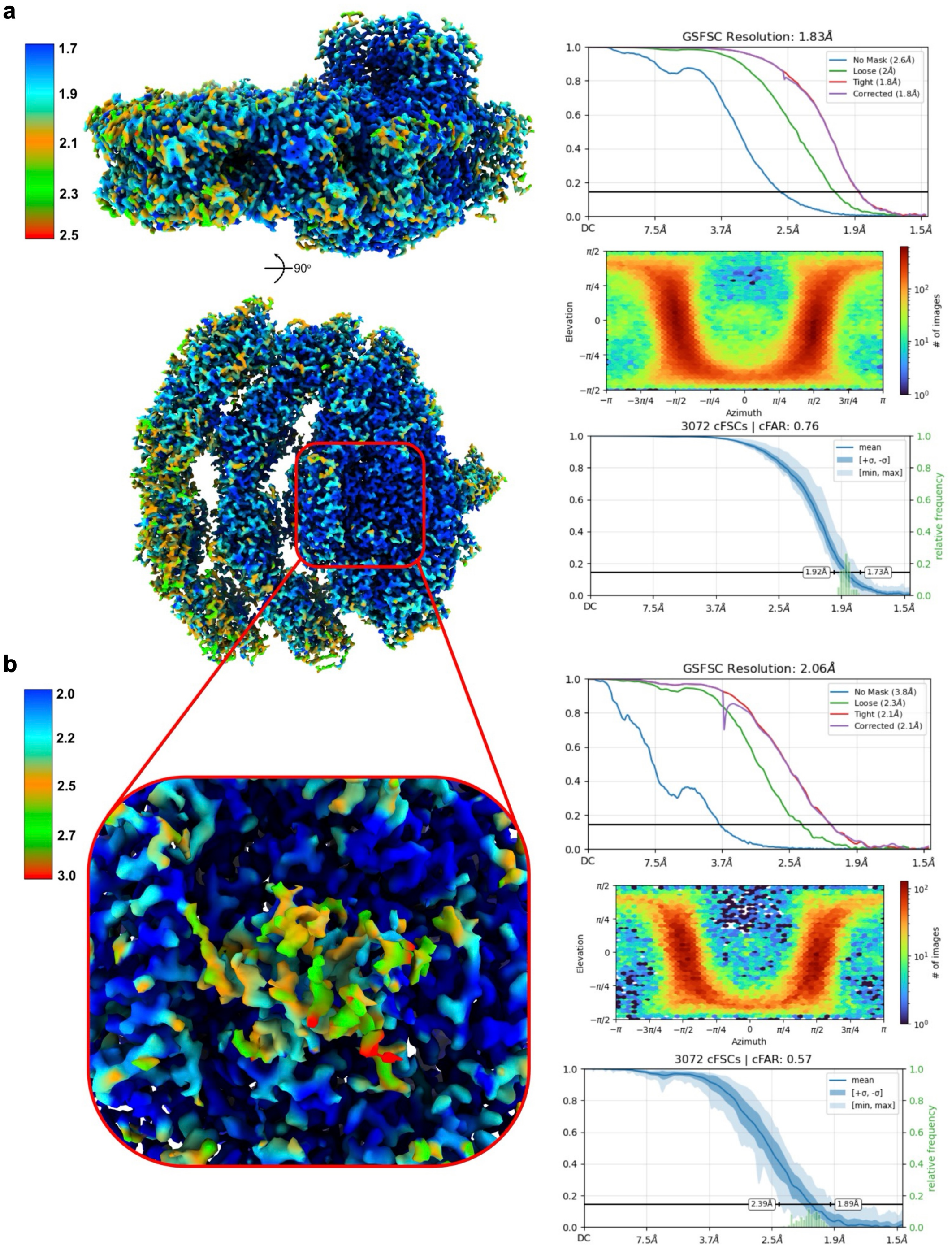

Supplementary Fig. 4. Cryo-EM maps resolution and quality. **a,** PSI cryo-EM map and **b,** Cyt c_6:PSI cryo-EM map colored by their local resolution calculated by GSFSC plot.

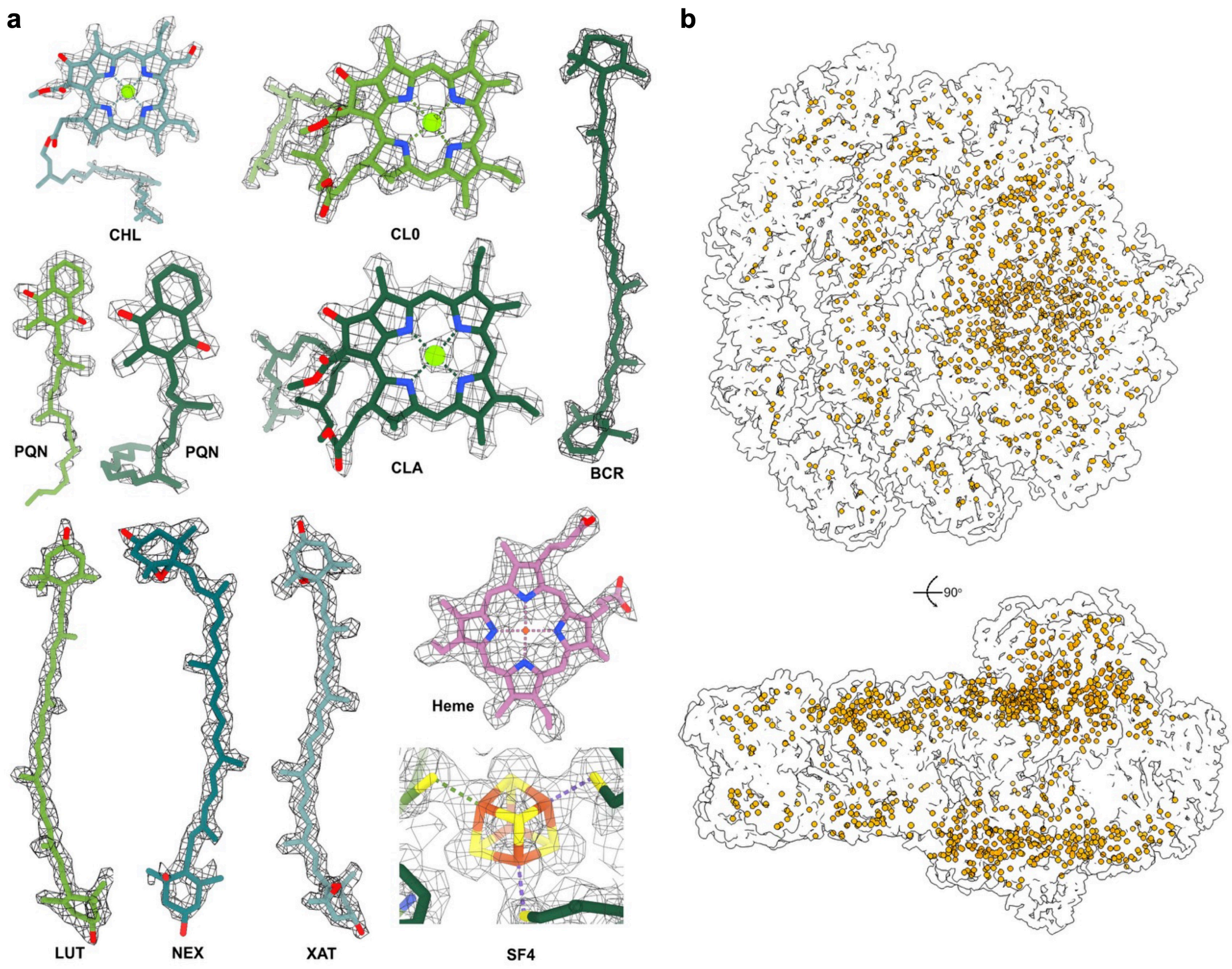

Supplementary Fig. 5. Electron-density maps of co-factors and water molecules distribution. **a**, Electron density maps of the co-factors: chlorophyll B (CHL), chlorophyll A isomer (CL0), chlorophyll A (CLA), Phylloquinone (PQN), Beta-Carotene (BCR), (3r,3'r,6s)-4,5-didehydro-5,6-dihydro-beta,beta-carotene-3,3'-diol (LUT), (1r,3r)-6-{(3e,5e,7e,9e,11e,13e,15e,17e)-18-[(1s,4r,6r)-4-hydroxy-2,2,6-trimethyl-7-oxabicyclo[4.1.0]hept-1-yl]-3,7,12,16 tetramethyloctadeca-1,3,5,7,9,11,13,15,17-nonaenylidene}-1,5,5-trimethylcyclohexane-1,3-diol (NEX), (3s,5r,6s,3's,5'r,6's)-5,6,5',6'-diepoxy-5,6,5',6'- tetrahydro-beta,beta-carotene-3,3'-diol (XAT), 4Fe4S iron/sulfur cluster (SF4). **b**, distribution of all the water molecules in orange.

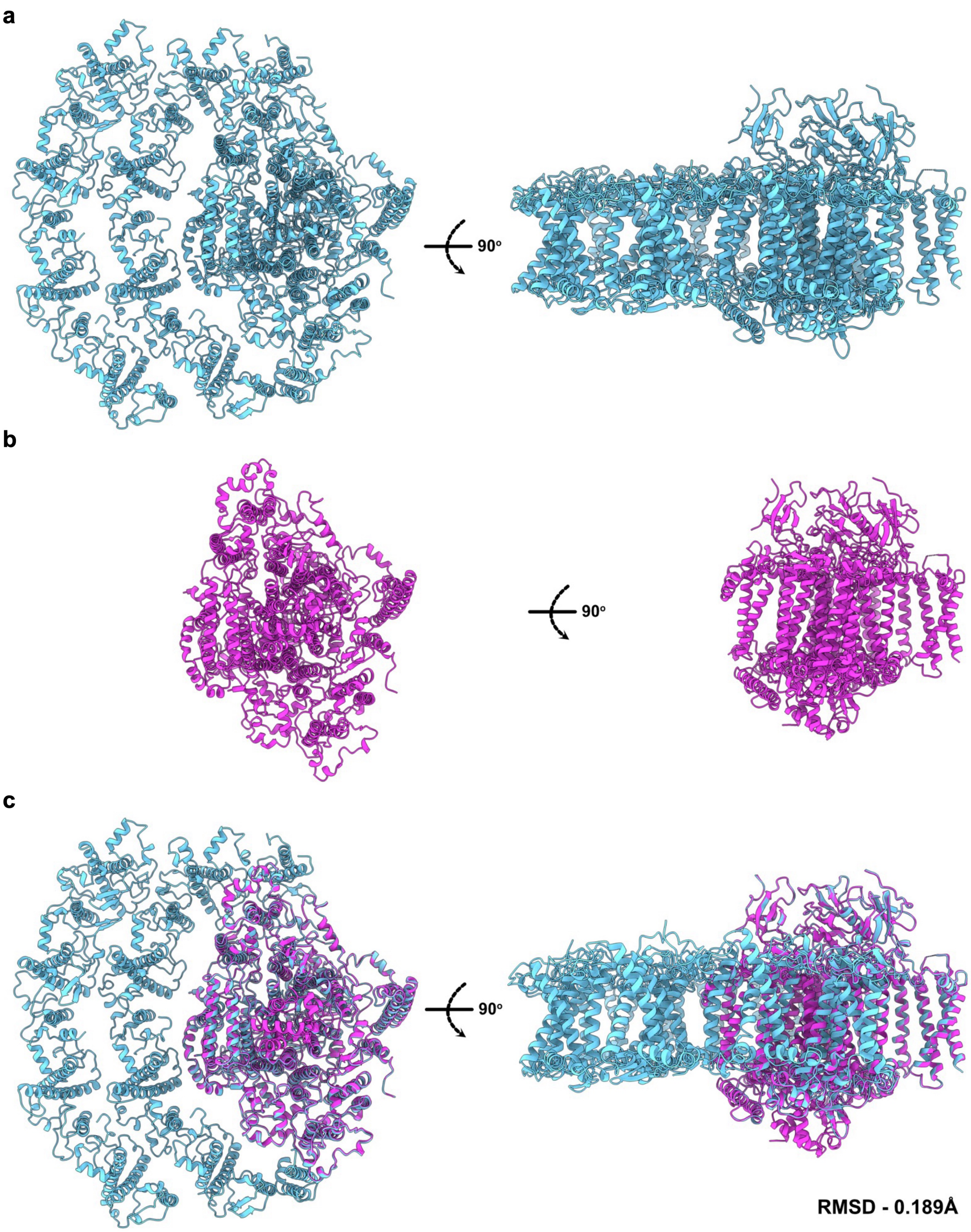

Supplementary Fig. 6. Structural comparison of PSI in the absence and presence of Cyt c_6. **a,** Atomic model of PSI particles lacking bound Cyt c_6, identified as a distinct population during single-particle analysis. **b,** Atomic model obtained from local refined cryo-EM map of Cyt c_6 bound to PSI. **c,** Structural superposition of the Cyt c_6-free PSI (sky) and Cyt c_6-bound PSI core (magenta). The two models align with a root-mean-square deviation (RMSD) of 0.189 Å, indicating that Cyt c_6 binding does not induce detectable conformational changes in the PSI core.

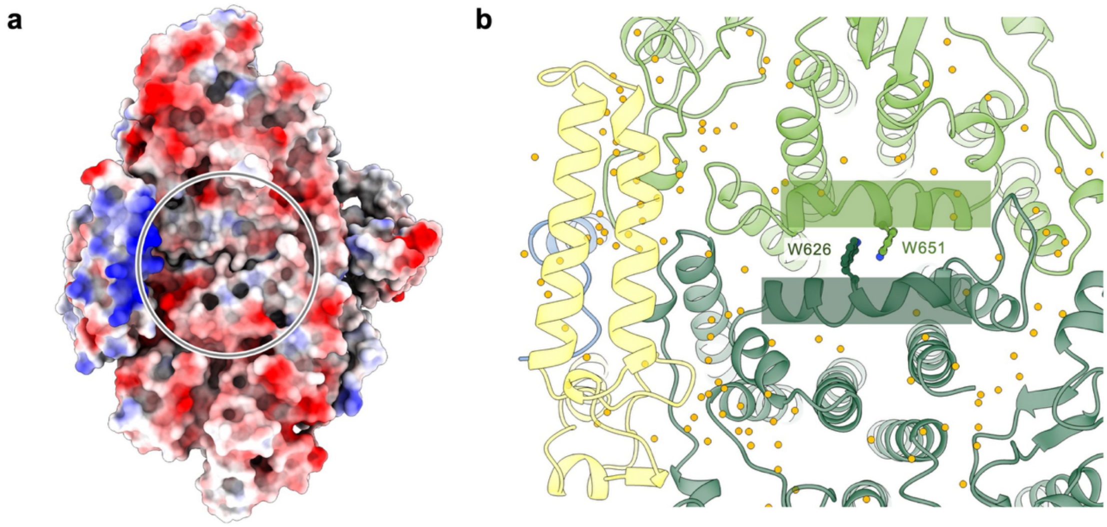

Supplementary Fig. 7. Luminal view of PSI core. **a,** Surface electrostatic potential distribution. Positively and negatively charged areas are in blue and red, respectively. Gray circle represents the shallow pocket. **b,** Cartoon representation with water molecules (orange dots). The Trp dimer is also shown. PsaA, PsaB and PsaF are colored in light green, dark green and yellow, respectively. Light green and dark green rectangles indicate PsaA-*l* loop and PsaB-*l'* loop, respectively.

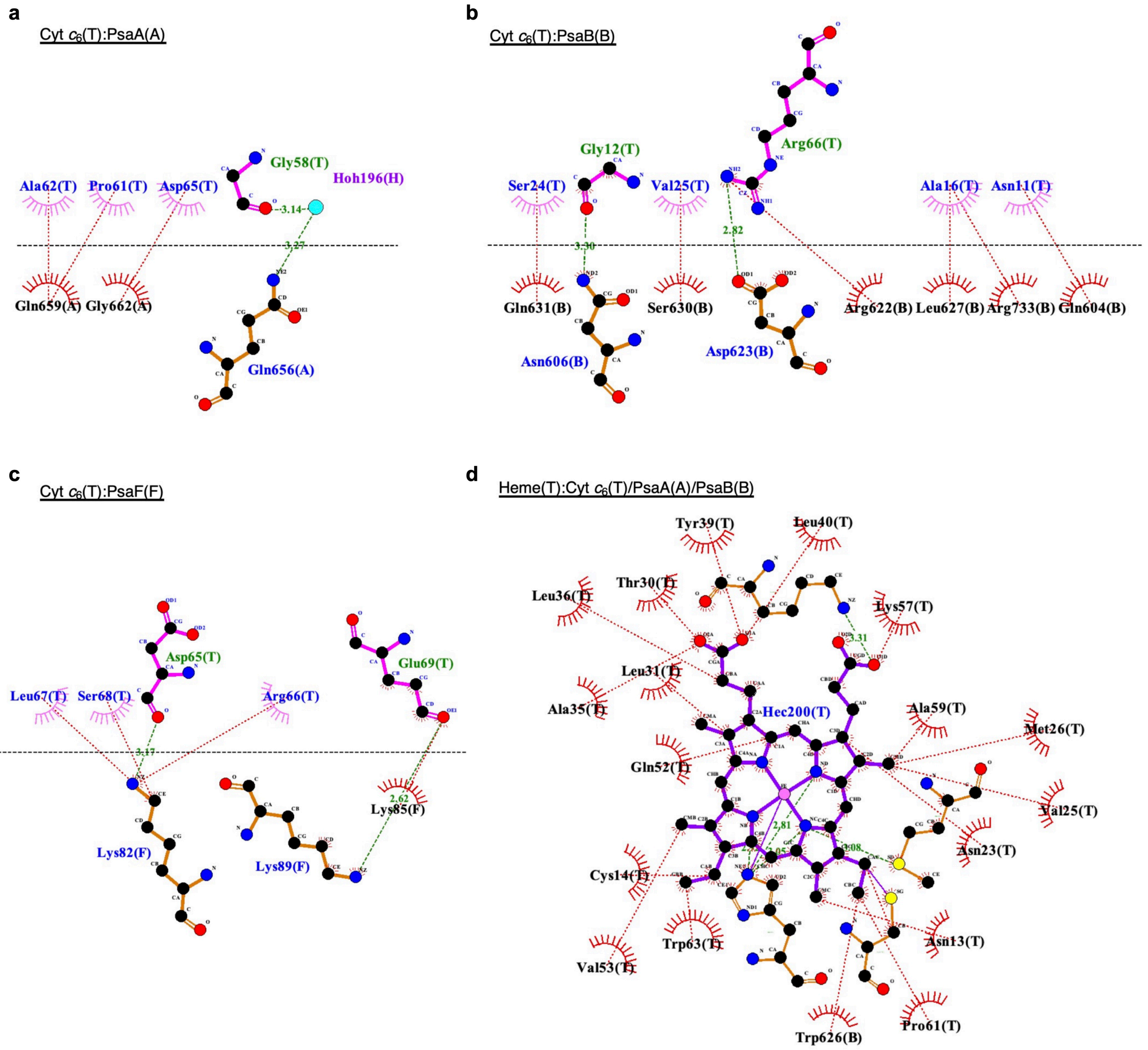

Supplementary Fig. 8. Protein(ligand)-protein interface diagrams of Cyt c_6:PsaA (**a**), Cyt c_6:PsaB (**b**), Cyt c_6:PsaF (**c**), and Heme:Cyt c_6/PsaA/PsaB (**d**), produced by LigPlot$^+$. Green dashed lines with numbers indicate potential hydrogen bonds or electrostatic interactions shorter than 3.35Å, providing the atom-atom distances (Å). Red dashed lines represent other non-bonded contacts including hydrophobic interactions.

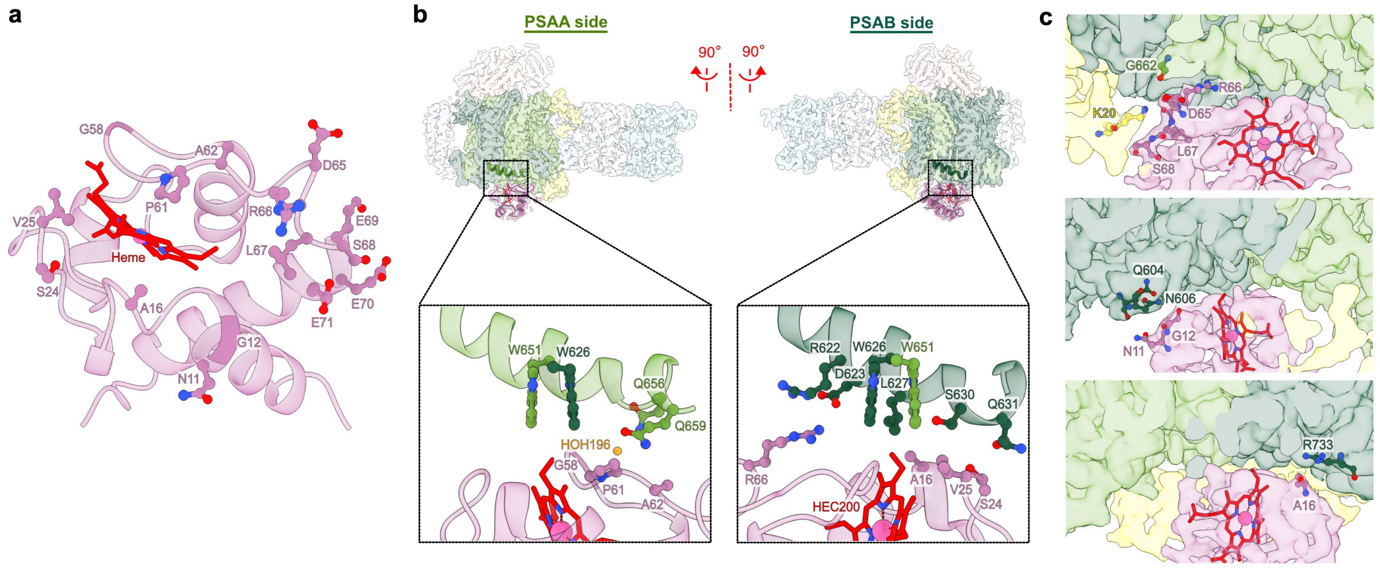

Supplementary Fig. 9. Additional figures for Cyt c_6:PSI binding. **a**, Stromal view of Cyt c_6:PSI interface (just Cyt c_6 side). Directly interacting amino acids are shown, in addition to E70 and E71. **b**, Interfaces between Cyt c_6 and PsaA-*l* loop (left)/PsaB-*l'* loop (right). Cyt c_6, PsaA, PsaB and PsaF are colored in pink, light green, dark green and yellow, respectively. Directly interacting residues and water molecules (orange) are shown in bottom panels, in addition to PsaA-W651. The Trp dimer is shown in both the bottom panels. **c**, Peripheral protein-protein interactions on the PsaA-PsaB shallow pocket.

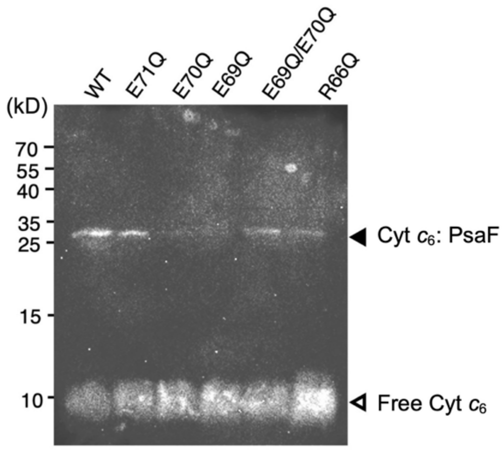

Supplementary Fig. 10. Cross-linking using Cyt c_6 variants with mutations at putatively interacting amino acid residues. The cross-linked samples were subjected to SDS-PAGE and Western blotting, where anti-Cyt c_6 antibodies were used. The same cross-linking experiments were performed three times, and a representative result is shown. Source data are provided as a Source Data file.

	PSI (EMDB-54803) (PDB 9SE6)	PSI-Cyt C_6 (EMDB-54804) (PDB 9SE7)
**Data collection and processing**		
Magnification	165,000	165,000
Voltage (kV)	300	300
Electron exposure (e–/Å^2)	50	50
Defocus range (μm)	-0.8 to -2.0	-0.8 to -2.0
Pixel size (Å)	0.73	0.73
Symmetry imposed	C1	C1
Initial particle images (no.)	1,313,388	1,313,388
Final particle images (no.)	246,159	52,617
Map resolution (Å)	1.83	2.06
FSC threshold	0.143	0.143
Map resolution range (Å)	1.92-1.73	2.39-1.89
**Refinement**		
Initial model used (PDB code)	7ZQC	1CYI
Model resolution (Å)	1.83	2.03
FSC threshold	0.143	0.143
Model resolution range (Å)	1.81-1.92	2.03-2.36
Map sharpening *B* factor (Å^2)	33.4	30.4
Model composition		
Non-hydrogen atoms	48478	24300
Protein residues	3993	2218
Ligands	LHG: 14, LUT:16, DGD:1, XAT: 7, SF4: 3, CHL: 28, CLA:188, PQN: 2, LMU: 27, NEX: 2, CL0: 1, BCR: 27, LMG: 12	LHG: 3, LUT:2, DGD:1, SF4: 3, CLA: 90, PQN: 2, LMU: 3, HEC: 1, CL0: 1, BCR: 15, LMG: 5
B factors (Å^2)		
Protein	10.34	24.56
Ligand	11.97	25.76
R.m.s. deviations		
Bond lengths (Å)	0.005	0.004
Bond angles (°)	0.941	0.708
Validation		
MolProbity score	1.29	1.47
Clashscore	5.35	6.34
Poor rotamers (%)	0.99	1.23

Ramachandran plot		
Favored (%)	98.46	97.77
Allowed (%)	1.54	2.23
Disallowed (%)	0.00	0.00

Supplementary Table 1. Cryo-EM data collection, refinement, and validation statistics.

Primer names	Sequence 5' to 3'
R66Q_fw	GGCGGATCAGCTGAGCGAAGAGGAGATCCAGG
R66Q_rv	CTCAGCTGATCCGCCCACGCCGGCATTGC
E69Q_fw	CCTGAGCCAGGAGGAGATCCAGGCGGTTGC
E69Q_rv	TCCTCCTGGCTCAGGCGATCCGCCCACG
E70Q_fw	GAGCGAACAGGAGATCCAGGCGGTTGCTGAATACG
E70Q_rv	ATCTCCTGTTCGCTCAGGCGATCCGC
E71Q_fw	CGAAGAGCAGATCCAGGCGGTTGCTGAATACG
E71Q_rv	TGGATCTGCTCTTCGCTCAGGCGATCCG
E69Q/E70Q_fw	TGAGCCAGCAGGAGATCCAGGCGGTTGCTGA
E69Q/E70Q_rv	TCTCCTGCTGGCTCAGGCGATCCGCCCA

Supplementary Table 2. Primers used in this study.
