# Supplementary material for: Cryo-EM structure of Chlamydomonas reinhardtii Photosystem I complexed with cytochrome c6
Source: Nat Commun. 2026 Mar 27;17:3031. doi: 10.1038/s41467-026-70944-9 (PMC13036084; doi:10.1038/s41467-026-70944-9)
Supplement: Supplementary file 2 — Reporting Summary [file 41467_2026_70944_MOESM2_ESM.pdf]

nature portfolio

Corresponding author(s): Jan Michael Schuller, Michael Hippler

Last updated by author(s): Feb 24, 2026

Reporting Summary

Nature Portfolio wishes to improve the reproducibility of the work that we publish. This form provides structure for consistency and transparency in reporting. For further information on Nature Portfolio policies, see our Editorial Policies and the Editorial Policy Checklist.

Statistics

For all statistical analyses, confirm that the following items are present in the figure legend, table legend, main text, or Methods section.

n/a	Confirmed	
☐	☒	The exact sample size (*n*) for each experimental group/condition, given as a discrete number and unit of measurement
☐	☒	A statement on whether measurements were taken from distinct samples or whether the same sample was measured repeatedly
☐	☒	The statistical test(s) used AND whether they are one- or two-sided<br>*Only common tests should be described solely by name; describe more complex techniques in the Methods section.*
☒	☐	A description of all covariates tested
☒	☐	A description of any assumptions or corrections, such as tests of normality and adjustment for multiple comparisons
☐	☒	A full description of the statistical parameters including central tendency (e.g. means) or other basic estimates (e.g. regression coefficient) AND variation (e.g. standard deviation) or associated estimates of uncertainty (e.g. confidence intervals)
☐	☒	For null hypothesis testing, the test statistic (e.g. *F*, *t*, *r*) with confidence intervals, effect sizes, degrees of freedom and *P* value noted<br>*Give P values as exact values whenever suitable.*
☒	☐	For Bayesian analysis, information on the choice of priors and Markov chain Monte Carlo settings
☒	☐	For hierarchical and complex designs, identification of the appropriate level for tests and full reporting of outcomes
☒	☐	Estimates of effect sizes (e.g. Cohen's *d*, Pearson's *r*), indicating how they were calculated

Our web collection on statistics for biologists contains articles on many of the points above.

Software and code

Policy information about availability of computer code

Data collection	Cryo-EM data were collected on a Titan Krios G4 transmission electron microscope (Thermo Fisher Scientific) operated at 300 kV, equipped with a Selectris energy filter and a Falcon 4 direct electron detector. Movies were acquired in counting mode at a nominal magnification of 165,000×, yielding a calibrated pixel size of 0.73 Å. Kinetic data were collected using a Joliot-type spectrophotometer (JTS-150, Biologic) with the manufacturer-provided software.
Data analysis	Cryo-EM data processing was performed in CryoSPARC v4. Micrographs were gain-corrected, aligned, and dose-weighted using Patch Motion Correction, followed by contrast transfer function (CTF) estimation with Patch CTF. Particle picking, 2D and 3D classifications, and refinements were all carried out in CryoSPARC v4. Model building was performed in Coot v0.9.8.96, and real-space refinement in PHENIX v1.21.2. The Grade Web Server was used to generate CIF restraint files. Final figures were prepared using ChimeraX v1.11. All kinetic data were processed and analyzed using the provided Biologics software. Data fitting was performed in OriginPro 2024, and graphical representations were created in BioRender.

For manuscripts utilizing custom algorithms or software that are central to the research but not yet described in published literature, software must be made available to editors and reviewers. We strongly encourage code deposition in a community repository (e.g. GitHub). See the Nature Portfolio guidelines for submitting code & software for further information.

Data

Policy information about availability of data

All manuscripts must include a data availability statement. This statement should provide the following information, where applicable:

- Accession codes, unique identifiers, or web links for publicly available datasets
- A description of any restrictions on data availability
- For clinical datasets or third party data, please ensure that the statement adheres to our policy

Structural data produced in this work, including cryo-EM density map and refined atomic coordinates, have been deposited in the Electron Microscopy Data Bank and the Protein Data Bank under the accession codes: PSI (PDB-9SE6 and EMDB-54803) and PSI-Cyt c6 (PDB-9SE7 and EMDB-54804). Source data for the biochemical analysis in Fig 1a,b and 2f and Supplementary Fig.10 are provided in the source data files.

Research involving human participants, their data, or biological material

Policy information about studies with human participants or human data. See also policy information about sex, gender (identity/presentation), and sexual orientation and race, ethnicity and racism.

Reporting on sex and gender	N/A
Reporting on race, ethnicity, or other socially relevant groupings	N/A
Population characteristics	N/A
Recruitment	N/A
Ethics oversight	N/A

Note that full information on the approval of the study protocol must also be provided in the manuscript.

Field-specific reporting

Please select the one below that is the best fit for your research. If you are not sure, read the appropriate sections before making your selection.

☒ Life sciences ☐ Behavioural & social sciences ☐ Ecological, evolutionary & environmental sciences

For a reference copy of the document with all sections, see nature.com/documents/nr-reporting-summary-flat.pdf

Life sciences study design

All studies must disclose on these points even when the disclosure is negative.

Sample size	For cryoEM analysis, in total, 21,413 movies were collected in order to have sufficient number of PSI particles, which resulted in 3D reconstructions with a resolution of 1.83Å (PSI map) and 2.06Å (Cyt c6-PSI-core map).
Data exclusions	For cryo-EM analysis, only PSI particles were retained for high-resolution structure determination, while all other particles were excluded.
Replication	For the kinetic assay, three biological replicates from independent complex isolations were performed. No experimental replicates were conducted for single-particle cryo-EM, given the robustness and reproducibility of the technique.
Randomization	Gold-standard resolution estimates were obtained by dividing particle images into two independent half-sets, as described in the text.
Blinding	Blinding was not necessary for this study. Furthermore, kinetic experiments were repeated with three biological replicates, and all assays yielded reproducible results.

Reporting for specific materials, systems and methods

We require information from authors about some types of materials, experimental systems and methods used in many studies. Here, indicate whether each material, system or method listed is relevant to your study. If you are not sure if a list item applies to your research, read the appropriate section before selecting a response.

Materials & experimental systems

n/a	Involved in the study
☐	☒ Antibodies
☒	☐ Eukaryotic cell lines
☒	☐ Palaeontology and archaeology
☒	☐ Animals and other organisms
☒	☐ Clinical data
☒	☐ Dual use research of concern
☒	☐ Plants

Methods

n/a	Involved in the study
☒	☐ ChIP-seq
☒	☐ Flow cytometry
☒	☐ MRI-based neuroimaging

Antibodies

Antibodies used: Cyt c6

Validation: https://doi.org/10.1111/j.1751-1097.2008.00440.x

Plants

Seed stocks: *Report on the source of all seed stocks or other plant material used. If applicable, state the seed stock centre and catalogue number. If plant specimens were collected from the field, describe the collection location, date and sampling procedures.*

Novel plant genotypes: *Describe the methods by which all novel plant genotypes were produced. This includes those generated by transgenic approaches, gene editing, chemical/radiation-based mutagenesis and hybridization. For transgenic lines, describe the transformation method, the number of independent lines analyzed and the generation upon which experiments were performed. For gene-edited lines, describe the editor used, the endogenous sequence targeted for editing, the targeting guide RNA sequence (if applicable) and how the editor was applied.*

Authentication: *Describe any authentication procedures for each seed stock used or novel genotype generated. Describe any experiments used to assess the effect of a mutation and, where applicable, how potential secondary effects (e.g. second site T-DNA insertions, mosiacism, off-target gene editing) were examined.*
