# Supplementary material for: Cryo-EM structure of Chlamydomonas reinhardtii Photosystem I complexed with cytochrome c6
Source: Nat Commun. 2026 Mar 27;17:3031. doi: 10.1038/s41467-026-70944-9 (PMC13036084; doi:10.1038/s41467-026-70944-9)
Supplement: Supplementary file 4 — Source Data [file 41467_2026_70944_MOESM4_ESM.zip › Source Data/Source data for Supplementary Fig.10.pdf]

Extended Data Fig.8

Raw image for markers

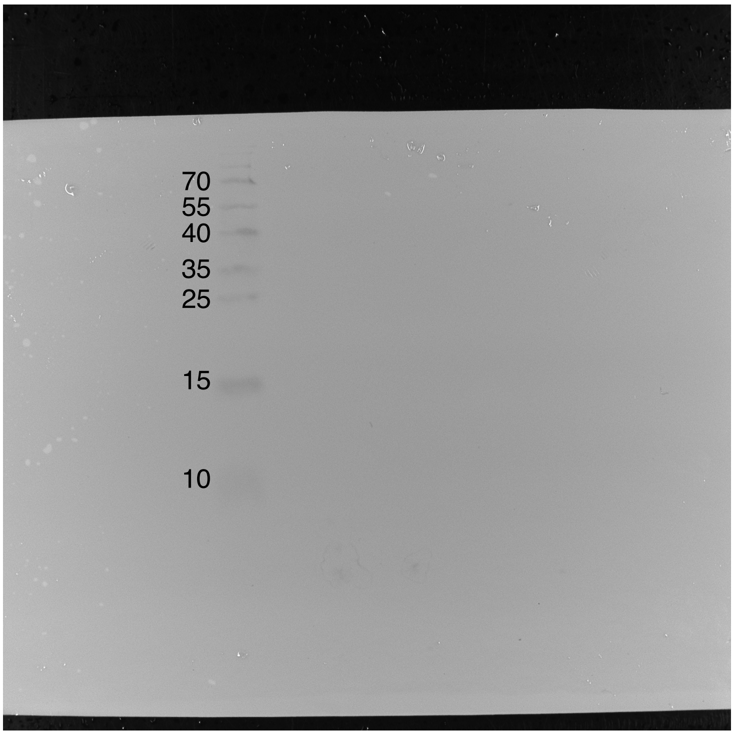

PageRuler™ Prestained Protein Ladder (Thermo Fischer Scientific) was used.

Blots (Luminescence)

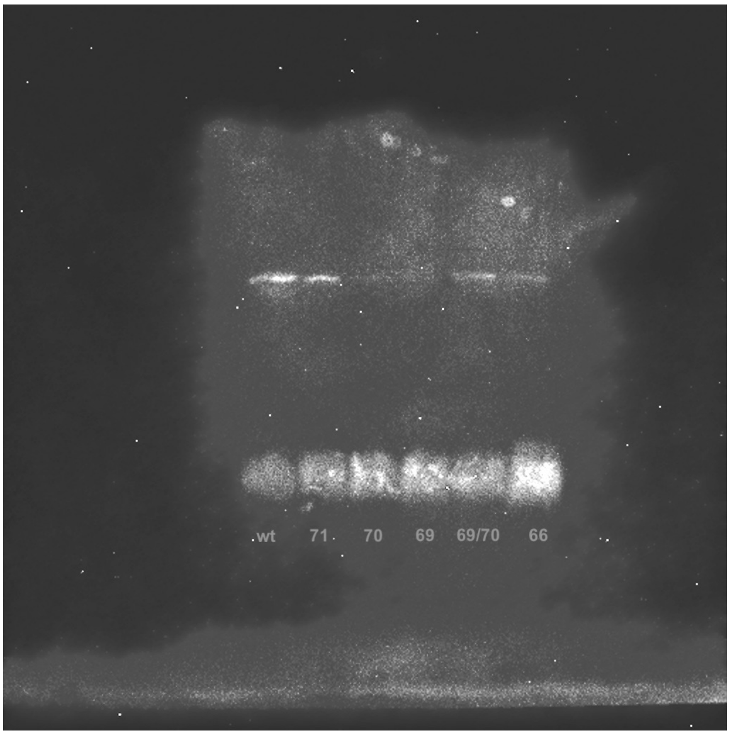

Anti-Cyt c_6 antibody, kindly provided by Prof. Sabeeha S.Merchant (University of California), was used.

The other 2 replicates

Raw image for markers

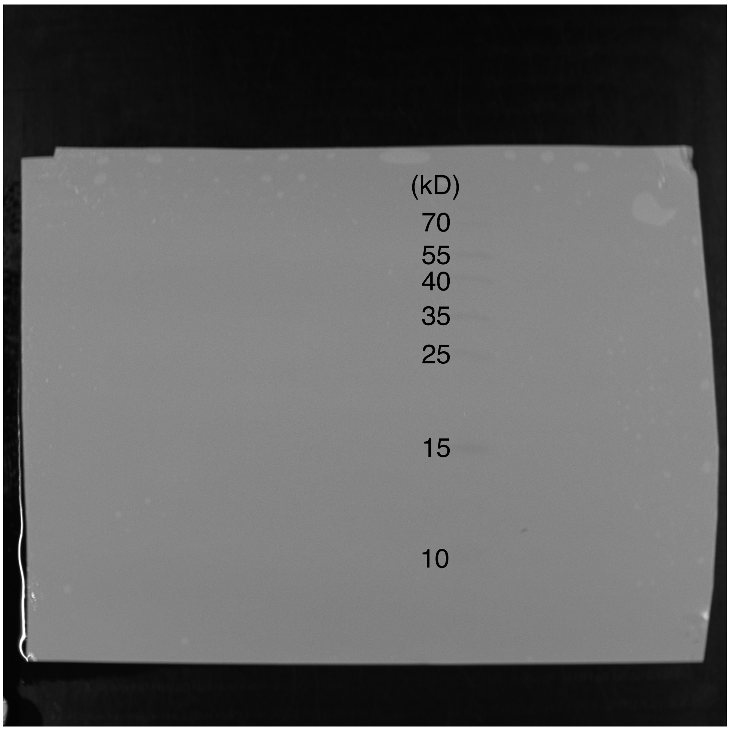

PageRuler™ Prestained Protein Ladder (Thermo Fischer Scientific) was used.

Blots (Luminescence)

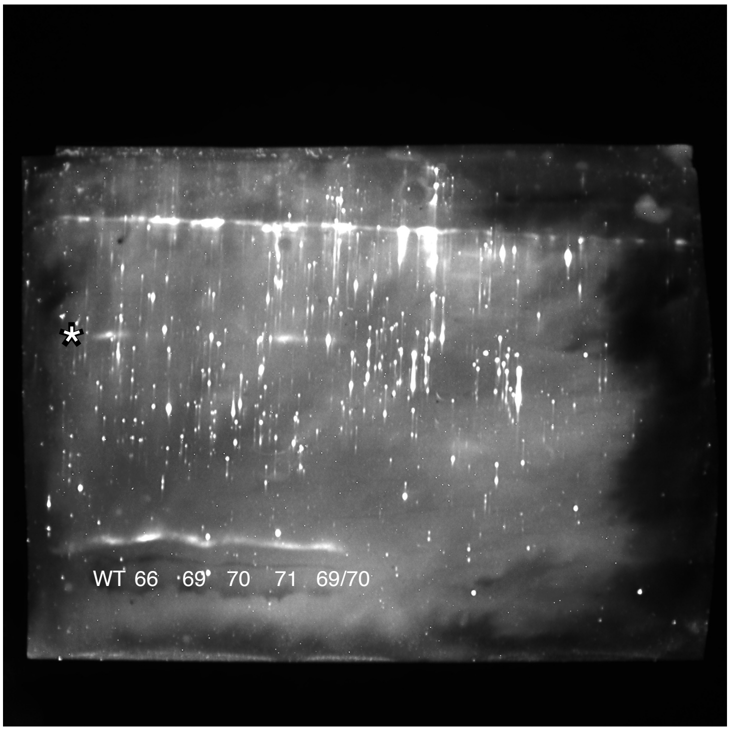

Anti-Cyt c_6 antibody, kindly provided by Prof. Sabeeha S.Merchant (University of California), was used.

*…cross-linked Cyt c_6: PsaF

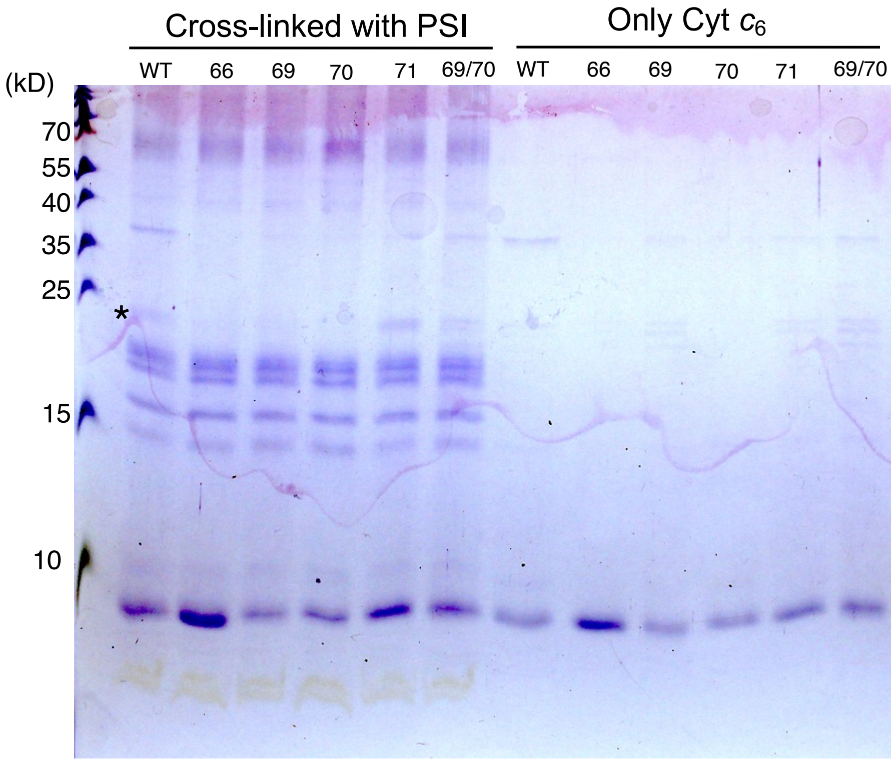

*…cross-linked Cyt c_6: PsaF
