# Supplementary material for: Cryo-EM structure of Chlamydomonas reinhardtii Photosystem I complexed with cytochrome c6
Source: Nat Commun. 2026 Mar 27;17:3031. doi: 10.1038/s41467-026-70944-9 (PMC13036084; doi:10.1038/s41467-026-70944-9)

Fig.1

a

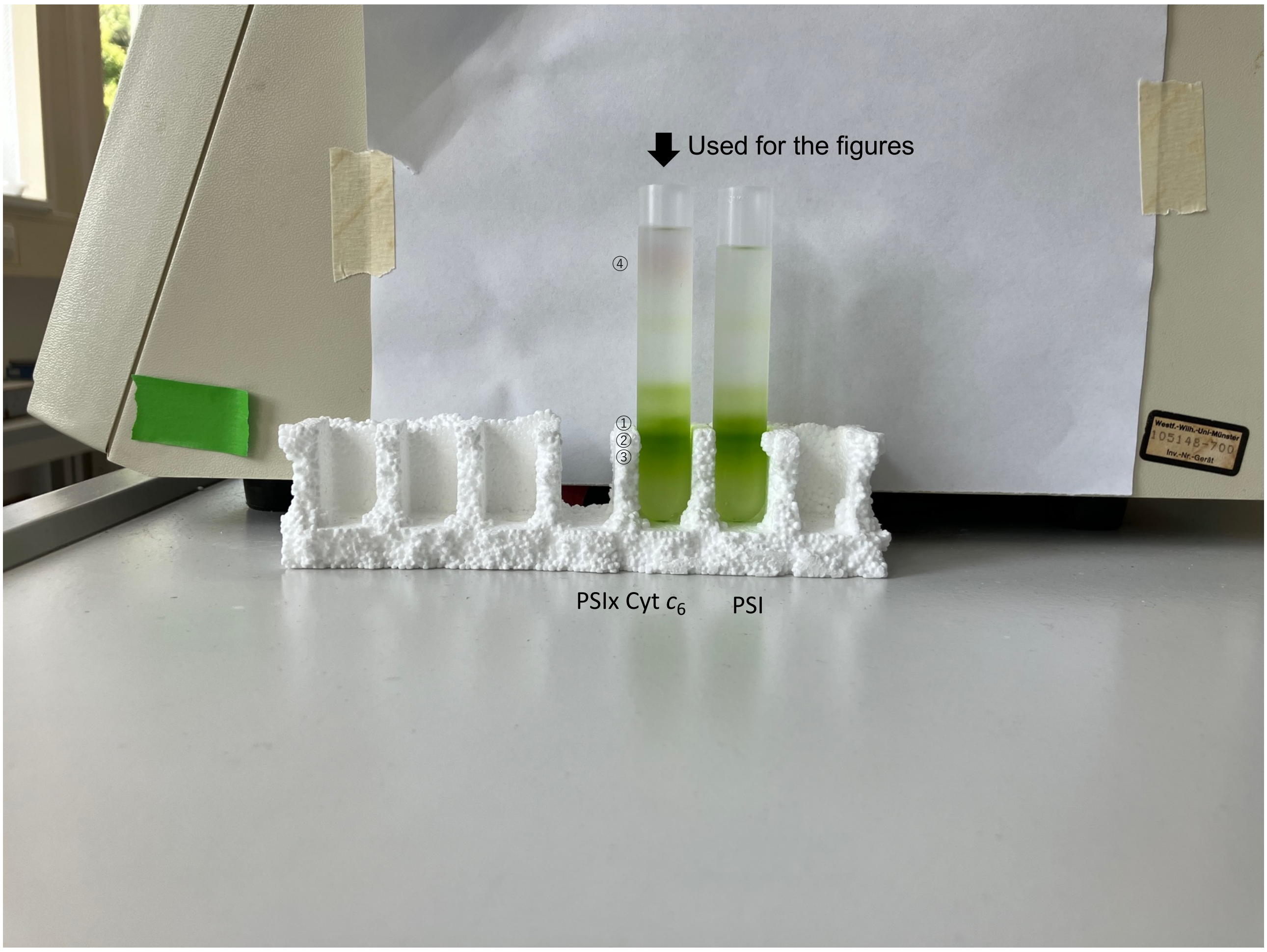

b

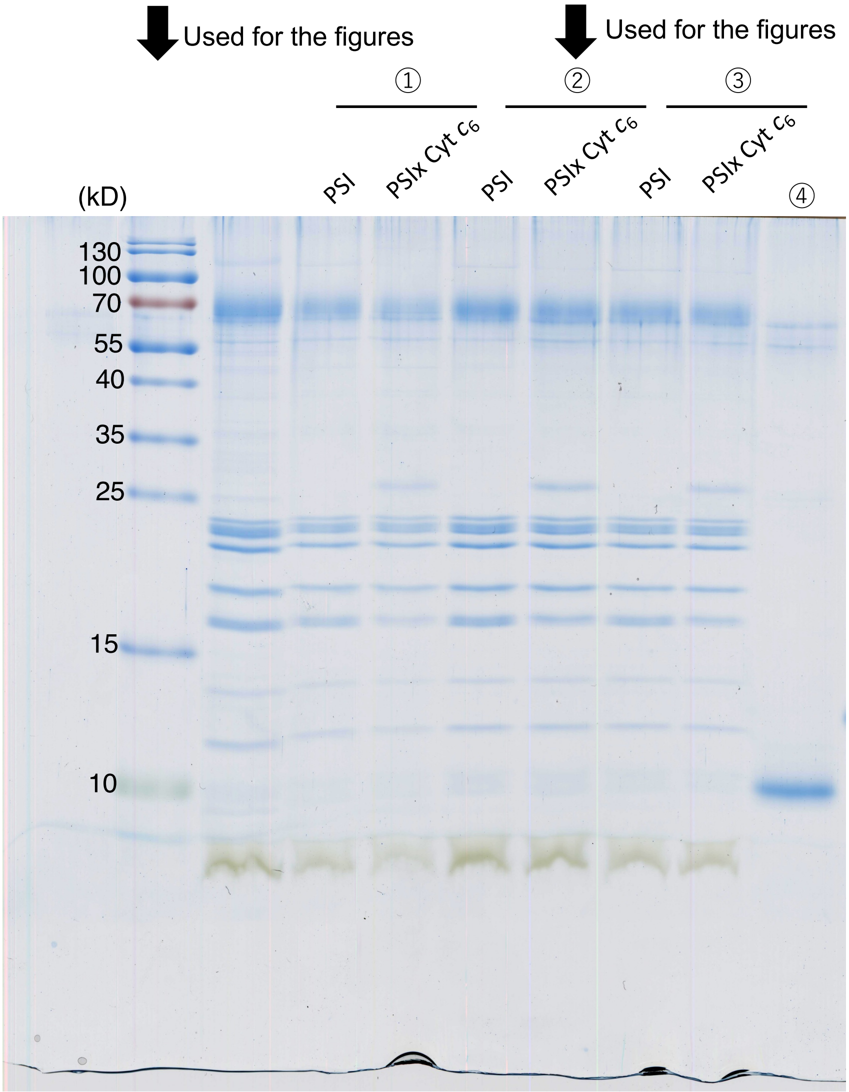

PageRuler™ Prestained Protein Ladder (Thermo Fischer Scientific) was used.

Other replicates

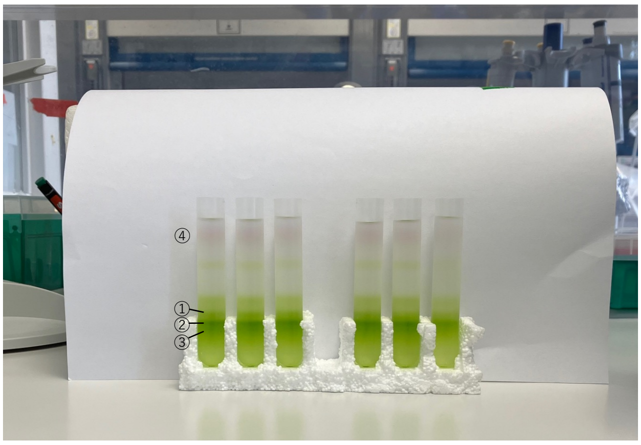

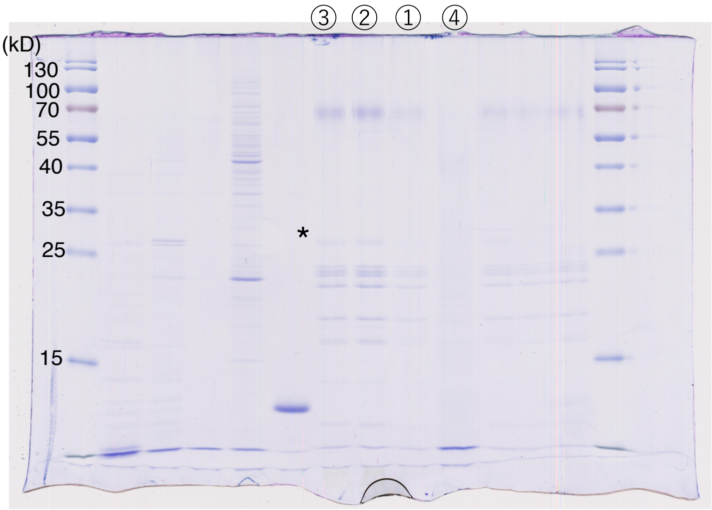

*...cross-linked Cyt c_6: PsaF

Cytochrome c_6	wt	wt	wt	wt	wt	wt	wt	wt	M
complex 2	PSI	PSI	PSI	PSI	PSI	PSI	PSI	-	
conc Cc_6 µM	0	1,25	2,5	5	10	20	40	40	

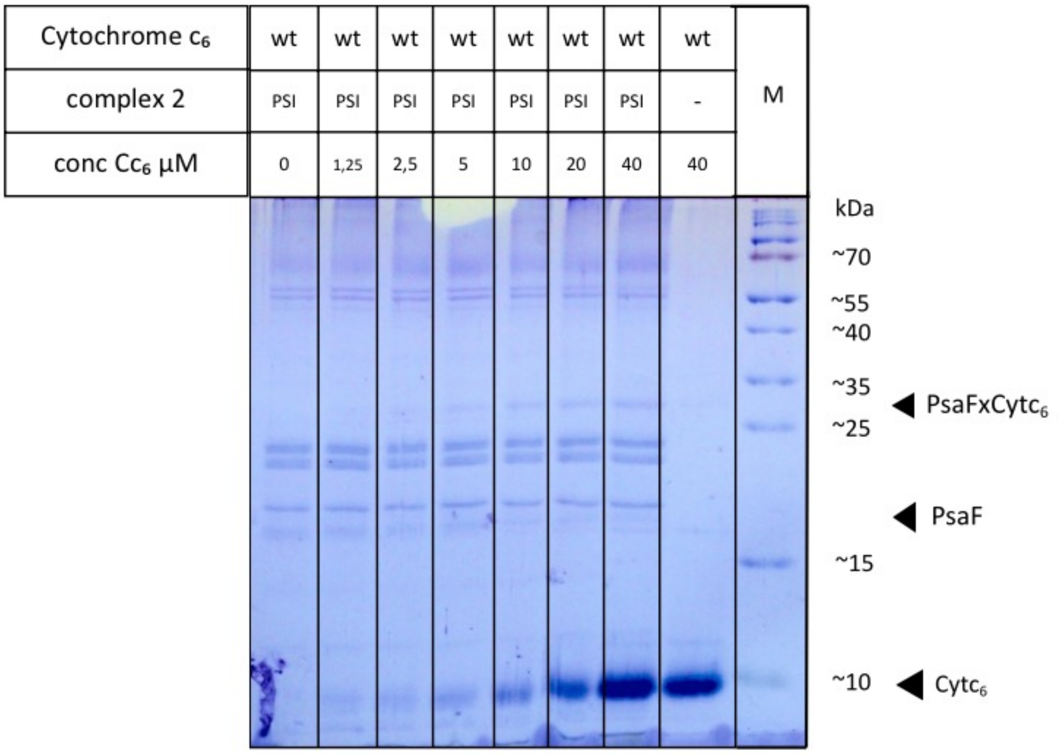

Supplement: Supplementary file 4 — Source Data [file 41467_2026_70944_MOESM4_ESM.zip › Source Data/Source data for Fig.1ab.pdf]
